# Precision phenotyping of elevated arousal in posttraumatic stress disorder: implications for personalized GABAergic pharmacotherapy

**DOI:** 10.3389/fpsyt.2026.1764293

**Published:** 2026-05-08

**Authors:** Vitaliana R. Vasquez, Jonathon R. Howlett

**Affiliations:** 1 VA San Diego Healthcare System, San Diego, CA, United States; 2 Department of Psychiatry, University of California San Diego, La Jolla, CA, United States

**Keywords:** anxious arousal, GABA, personalized medicine, posttraumatic stress disorder, psychopharmacology

## Abstract

Posttraumatic stress disorder (PTSD) is a common and disabling condition, and current first-line medications have remission rates as low as 20-30%. Novel agents targeting the GABAergic system offer a promising alternative approach to treatment given converging evidence for a central role for this system in PTSD pathophysiology. However, overall progress in PTSD psychopharmacology has been hampered by the substantial heterogeneity in this population, which has been highlighted by recent research on both subjective symptoms and underlying biological mechanisms. In this mini review, we examine the literature on heterogeneity in PTSD, particularly with respect to anxious arousal. We examine heterogeneity in arousal based on both clinical symptoms and objective behavioral or biological markers. We also examine evidence for a specific role for GABAergic dysfunction in causing elevated anxious arousal. These lines of research suggest that novel GABAergic agents may be particularly effective in a high arousal PTSD subgroup. Future research should incorporate individual subjective and objective measures of anxious arousal into clinical trials of GABAergic agents for PTSD, with the ultimate aim of developing personalized treatments for selected individuals within this population.

## Introduction

First-line medications for posttraumatic stress disorder (PTSD) have remission rates as low as 20-30% (1), causing leaders in the field to describe the lack of advancement in PTSD psychopharmacology as an urgent crisis (2). One highly promising avenue is the development of novel agents targeting the GABAergic system, in light of converging evidence for a central role of this system in PTSD pathophysiology (3). If effective, such agents have the potential to transform PTSD psychopharmacology for individuals who do not respond or remit with currently available therapies.

Despite the promise of novel GABAergic agents in PTSD, progress in PTSD psychopharmacology continues to be hampered by marked heterogeneity within this population. Patterns of DSM symptoms vary substantially across individuals, with a recent analysis of data from the Army Study to Assess Risk and Resilience in Servicemembers (Army STARRS) finding that, of 3511 participants with a PTSD diagnosis, there were 2181 different symptom patterns, and 55% of individuals exhibited a pattern of symptoms which was unique to that individual (4). Similarly, studies of mechanisms in PTSD have revealed divergent biological markers across subgroups, ranging from exaggerated to blunted arousal (5). Precision phenotyping of mechanistic subgroups within PTSD could enable personalized treatment with novel therapies, such as GABAergic agents, improving outcomes for individual patients.

In this mini review, we summarize the literature on heterogeneity in PTSD with an emphasis on evidence for a high arousal subgroup. We review arousal-related heterogeneity based on both clinical symptoms and objective behavioral or biological markers (focusing on individual differences within patients rather than between patients and healthy controls). We also examine evidence for a specific role for GABAergic dysfunction in causing elevated anxious arousal. Taken together, these emerging lines of research suggest that novel GABAergic agents may be particularly effective in high arousal PTSD. We conclude with a discussion of the implications for personalized pharmacotherapy of elevated arousal in this population.

## Heterogeneity in Anxious Arousal in PTSD

Analyses based on self-report measures have found that PTSD is characterized by prominent general distress as well as features of anxious arousal (6, 7), raising the possibility that separate affective dimensions may vary in intensity across individual patients. Consistent with this, research highlights elevations in arousal-related symptoms as a distinct PTSD presentation. For example, a latent class analysis (LCA) applied to 594 military personnel found three classes of traumatic symptoms, including a class characterized by low overall symptom burden, a class characterized by severe trauma symptoms coupled with tension-reduction behaviors, and a class characterized by increased arousal (8). The arousal class was characterized by intermediate levels of overall severity in depression and anxiety symptoms as well as by increased blast exposure compared to the low symptom burden class. Another study applying LCA to traumatic stress reactions in 3,727 U.S. military veterans found evidence for 5 classes of veterans, including classes with low traumatic stress reactions, high traumatic stress reactions, high levels of depression and anxiety symptoms, high avoidant arousal, and high dysphoric arousal (9). The avoidant arousal and dysphoric arousal classes comprised 9.2% and 8.2% of the sample, respectively. The dysphoric arousal class, along with the high traumatic stress reaction class, was characterized by lower levels of functioning. Taken together, these studies suggest that traumatic reactions characterized by elevations in arousal are associated with significant symptom burden and dysfunction and may warrant special attention for treatment.

Some studies have examined the possibility that differences in trauma exposure may partially account for heterogeneity in subsequent PTSD symptoms. For example, a cross-sectional study of 367 mental health outpatients with trauma exposure found that combat and sexual trauma were associated with worse total PTSD severity (10). Combat trauma was specifically associated with elevations in arousal and intrusions, while sexual trauma was associated with conscious avoidance and negative cognitions and mood. Another study of 3,507 U.S. veterans found that interpersonal violence, combat, and captivity were associated with more severe PTSD symptoms, including dysphoric arousal but also intrusive thoughts, avoidance, negative affect, anhedonia, and externalizing behaviors (11). Other research has also found that exposure to physical and sexual violence is associated with overall PTSD symptom severity (12, 13). Thus, while trauma type consistently predicts overall PTSD severity, evidence for an association between trauma type and a specific high arousal presentation is mixed, suggesting that other factors may drive the emergence of this presentation in individual patients.

Arousal-related peripheral and neural markers have been used to investigate heterogeneity in PTSD and related disorders. A study of 49 individuals with PTSD and 76 control participants with varying levels of trauma severity found that PTSD was associated with increased physiological reactivity, characterized by increased startle reflex, autonomic response, and facial expressivity during trauma imagery (14). However, PTSD patients with greater trauma exposure displayed blunted physiological activity, despite reporting higher subjective arousal. These findings suggest that objective markers of arousal may reveal mechanistic differences that are not apparent from self-reported arousal symptoms. Similar findings were reported in a different study of 54 trauma-exposed college students, which found that moderate trauma exposure and moderate PTSD symptoms were associated with exaggerated autonomic responses to acoustic startle, while severe trauma exposure and severe PTSD symptoms were associated with blunted autonomic responses (15). A structural magnetic resonance imaging (MRI) study in 48 Iraq and Afghanistan combat veterans found that reduced right amygdala volume was most strongly associated with anxious arousal symptoms (16). Additionally, right amygdala volume fully mediated the relationship between severity of combat exposure and anxious arousal, suggesting that this neural marker may reflect a causal mechanism of the influence of trauma on affect. Another study of 16 individuals with PTSD and 32 healthy controls found that greater activation of the brainstem, thalamus, amygdala, and medial prefrontal cortex in response to fearful faces was associated with concurrent autonomic reactivity to the same fearful faces (17), suggesting that individual differences in arousal response may be driven by measurable differences in neural processing. A study of 423 individuals with probable PTSD, from a prospective study of U.S. Army soldiers deployed to Afghanistan, found that a threat-reactivity subgroup (identified using latent profile analysis of PTSD symptoms) exhibited lower polygenic risk for major depression compared with a distinct dysphoric subgroup (18), suggesting there is a genetic component to arousal-related heterogeneity in PTSD. Taken together, these studies suggest that there is substantial variability in peripheral measures of arousal within individuals with PTSD, ranging from exaggerated to blunted responses, and that this variability might partly depend on the level of trauma exposure and on genetic factors. Distinct patterns of objective and subjective arousal may also be underpinned by differences in brain structure and function.

## GABA receptor modulation as a mechanism to target anxious arousal in PTSD

GABA is the primary inhibitory neurotransmitter in the brain and plays a critical role in the balance of excitation and inhibition in neural networks (19). A wealth of converging evidence specifically implicates GABA signaling as a crucial regulator of arousal. A wide range of central nervous system depressants, which reduce arousal, act via GABAergic mechanisms (20). The GABA_A_ receptor modulators benzodiazepines robustly reduce acute arousal, displaying clear sedative and anxiolytic effects. They have well documented efficacy for anxiety disorders, with extensive evidence for the treatment of panic disorder (21). However, benzodiazepines do not have an accepted role in the treatment of depression, and research indicates they may be more effective for somatic symptoms of anxiety compared to “psychic anxiety”, e.g. worry (22). This suggests that benzodiazepines may have relatively specific effects in reducing anxious arousal rather than reducing general distress or increasing positive affect. While benzodiazepines and other positive GABA modulators are anxiolytic, negative GABA modulators are anxiogenic, reinforcing the relationship between GABA signaling and anxious arousal (23).

In addition to causal evidence based on pharmacologic manipulation, a range of correlational evidence implicates GABAergic signaling in anxiety and PTSD (3). A review of fourteen positron emission tomography (PET) studies in anxiety disorders found evidence for reduction in GABA_A_ receptor binding across the mesolimbocortical system (24). PET studies have also found evidence for reduced GABA_A_ receptor binding specifically in panic disorder and PTSD (25, 26). Magnetic resonance spectroscopy (MRS) has revealed a significant reduction in GABA in the insula in PTSD, as well as a negative relationship between GABA and state and trait anxiety (27). Examination of gene expression in post-mortem brain tissue has revealed an association between PTSD and downregulation of a set of interconnected transcripts expressed in inhibitory GABAergic interneurons, consistent with decreased GABAergic transmission in PTSD (28). Reductions in GABA signaling associated with anxious arousal may be related to reductions in GABA concentration, GABA receptor density, interneuron activity, altered subunit compositions of GABA receptors, and altered endogenous modulation of GABA receptors by stress-related neurosteroids (29). Finally, relative GABA signaling deficiencies could be related to excessive activity of the excitatory glutamatergic system in anxiety and PTSD, causing an imbalance of neuronal excitation and inhibition that could be corrected via enhanced GABAergic signaling (30).

It is generally believed that the anxiolytic effect of positive GABA modulation is mediated by actions on a specific network of arousal-related neural regions (31). Rodent research combining imaging, electrophysiology, and opto- and chemo-genetic manipulation has identified the amygdala as a central mediator of the anxiolytic effect of benzodiazepines (32). While not conclusive, human research suggests that benzodiazepine effects may additionally be mediated by effects on the insula and other cortical regions (33). Two fMRI studies demonstrate that the benzodiazepine lorazepam causes a dose-dependent reduction in insula and amygdala activity during emotion processing and risky decision-making (34, 35).

Based on a small number of small clinical trials, benzodiazepines have not been shown to be effective treatments for PTSD (36). This may be due to heterogeneity in this population or to the development of tolerance to benzodiazepine effects, eroding efficacy during long-term treatment. Clinical practice guidelines for PTSD recommend against the use of benzodiazepines to treat PTSD given the lack of evidence for efficacy as well as the evidence for long-term risks, which include misuse and cognitive changes (21, 37).

The risks of long-term benzodiazepine use, as well as reduced long-term efficacy due to tolerance, have led to a search for alternative agents which may correct deficient GABA signaling without the downsides of benzodiazepines (see **Table 1**). One major class of agents under development is the selective GABA_A_ receptor modulators, which target specific subtypes of GABA_A_ receptors (39). Different subtypes of GABA_A_ receptors are selectively expressed in different neural regions and display diverse functional effects. They may therefore separately mediate the sedative, amnestic, anticonvulsant, analgesic, and anxiolytic effects of benzodiazepines. Pooled analysis of phase 1 clinical trials in humans has shown that α2-selective GABA_A_ modulators display relatively specific effects in reducing peak saccade velocity (a sensitive marker of GABA function), with reduced effects on alertness and cognition compared to benzodiazepines (40).

**Table 1:** Medications Affecting GABA Signaling with Potential Relevance to PTSD.

Medication Class	Medication	Primary Therapeutic Target	Putative GABAergic Effect
Neurosteroids	PRAX-114	Extrasynaptic-preferring positive allosteric modulation of GABA_A_ receptors	Direct GABA receptor action
	Brexanolone	Positive allosteric modulation of GABA_A_ receptors	Direct GABA receptor action
	Ganaxolone	Positive allosteric modulation of GABA_A_ receptors	Direct GABA receptor action
Acetylcholine Receptor Modulators	BNC-210	Negative allosteric modulation of α7 nicotinic acetylcholine receptor	Modulation of GABAergic tone via acetylcholine receptors on GABA interneurons
GABA Reuptake Inhibitors	Tiagabine	Selective blockade of GABA transporter	Modulation of GABAergic tone via reuptake inhibition
Cannabinoid Modulators	Cannabidiol	Activation of the endogenous endocannabinoid system	Modulation of GABA_A_ receptors and GABAergic interneurons
	JZP-150	Inhibition of fatty acid amide hydrolase (FAAH)	Modulation of GABAergic tone via cannabinoid receptors on GABAergic interneurons
Gabapentinoids	Pregabalin	Inhibition of release of excitatory neurotransmitters, including glutamate, via voltage gated calcium channels	Indirect modulation of GABAergic tone via inhibition of excitatory signaling
	Gabapentin	Inhibition of release of excitatory neurotransmitters, including glutamate, via voltage gated calcium channels	Indirect modulation of GABAergic tone via inhibition of excitatory signaling
Glutamatergic Agents	Ketamine	Noncompetitive antagonism of N-methyl-D-aspartate (NMDA) receptor	Indirect modulation of GABAergic tone via glutamatergic signaling
	Lamotrigine	Inhibition of voltage-gated sodium channels	Modulates balance of excitation and inhibition by suppressing glutamate release
	Riluzole	Inhibition of voltage-gated sodium channels	Believed to directly or indirectly modulate glutamatergic and GABAergic signaling
	Topiramate	Modulation of AMPA/Kainate, GABA_A_, and voltage-gated sodium channels	Direct modulation of GABA receptors as well as modulation of excitatory signaling

Table was adapted in part from Norred, et al. (38).

Several other classes of potential novel anxiolytics act directly or indirectly to modulate GABA signaling. Neurosteroids, such as brexanolone (currently FDA-approved for postpartum depression), ganaxolone, and the novel agent PRAX-114, act as positive endogenous modulators of GABA_A_ receptors (38, 41). Research indicates that neurosteroids exhibit rapid anxiolytic effects but appear to have a lower propensity for tolerance compared to benzodiazepines (42). A phase 2 trial of PRAX-114 for PTSD was initiated (NCT05260541) but terminated, while a phase 2 trial of ganaxalone for PTSD found no difference between ganaxolone and placebo (43), and a planned phase 2 dose-finding trial of brexanolone for concurrent PTSD and stress-induced alcohol use is not yet recruiting (NCT06580444). Other classes of potential novel anxiolytics include cannabinoids (such as cannabidiol (CBD)) and modulators of the endocannabinoid system (such as fatty acid amide hydrolase (FAAH) inhibitors). While cannabinoid receptors are present on a number of different cell types in the central nervous system, cannabinoid receptors on glutamatergic and GABAergic neurons appear to be particularly important in mediating effects on anxiety (44). Furthermore, both CBD and the major central endocannabinoid 2-arachidonoyl glycerol (2-AG) act directly as positive modulators of GABA_A_ receptors (45). Two trials of CBD for PTSD are currently recruiting (NCT04550377 and NCT05269459), while a trial of the FAAH inhibitor JZP-150 for PTSD did not meet its primary endpoint (NCT05178316). The gabapentinoids pregabalin and gabapentin, while not acting directly at GABA_A_ receptors, are thought to modulate GABAergic tone indirectly via inhibition of excitatory signaling (46). The GABA reuptake inhibitor tiagabine did not improve PTSD symptoms compared to placebo in a randomized controlled trial (47). The novel agent BNC-210, a negative allosteric modulator of the α7 nicotinic acetylcholine receptor, is believed to modulate GABAergic tone via acetylcholine receptors on GABA interneurons (38). A phase 2 trial of BNC-210 found that it significantly improved PTSD symptoms (48). Finally, a number of potential agents act as modulators of the glutamatergic system, including medications approved for other indications (such as ketamine, lamotrigine, riluzole, and topiramate) and novel agents under development (30). As the major excitatory neurotransmitter in the central nervous system, glutamate is critical in determining the balance of excitation and inhibition in neural circuits. Ketamine has been shown to rapidly normalize stress-induced impairments in GABA signaling in animal models (49), suggesting that measures of arousal-related dysfunction in the GABA system may be helpful for targeting glutamatergic medications. See Norred et al. (38) for a recent review of novel pharmacologic approaches to PTSD.

GABA modulation may interact with other biological systems, including inflammatory responses and the hypothalamic-pituitary adrenal (HPA) axis (50). By stimulating the HPA axis, stress can affect the immune system, increasing risk of hypercortisolism and potentially contributing to PTSD symptoms. Additionally, the activation of inflammasomes such as NLRP3, which drives proinflammatory cytokines, may contribute to PTSD symptoms (50). Glutamate stimulates corticotropin-releasing hormone (CRH), while GABA regulates CRH neurons (50). Restoring GABAergic tone may thereby normalize HPA-related dysfunction in PTSD.

## Discussion

The literature reviewed here suggests that: 1) a subgroup of individuals with PTSD displays a relatively specific increase in anxious arousal; 2) novel positive GABA receptor modulators may be particularly effective for elevated anxious arousal. Thus, a personalized approach to treatment in which positive GABA receptor modulators are targeted to individuals in this high arousal subgroup may improve treatment outcomes. Such a personalized approach may help overcome the limitations of previous clinical trials (which have tended to have mixed results) and to enable improved treatment of individuals with a variety of comorbidities (by focusing treatment on mechanistic dysfunction rather than categorical diagnoses). However, these hypotheses remain to be demonstrated in clinical trials.

A critical consideration for personalized medicine is the measurement reliability of criteria for subgroup determinations that drive treatment decisions. It is significantly more challenging to develop an assay which provides clinically useful individualized measurements, compared to assays which only detect average differences between groups (51). In particular, objective markers to support individualized clinical decision making require excellent measurement reliability, with internal consistency ideally being greater than 0.9 (52), while many behavioral and biological markers of psychopathological mechanisms fall well short of this criterion. Recent analyses have highlighted low measurement reliability in a wide range of behavioral measures, with median intraclass correlation coefficient (ICC) of.311 for behavioral markers of self-regulation (53). Biomarkers derived from functional neuroimaging also typically exhibit relatively poor reliability, with the mean ICC of common fMRI measures being .397 (54). Similarly, the results of both task-based and resting state functional imaging studies in PTSD have tended to be inconsistent across studies, reducing the usefulness of imaging markers for advancing clinical research and personalized treatment (55).

A number of strategies have been proposed to address the reliability and validity of behavioral measures (51). One promising strategy to increase reliability is a focus on temporally dense data collection during real-time behavioral tasks (56). By increasing the number of data points collected, this approach can improve the reliability of measures derived from these data points. For example, joysticks (57) or smartphone sensors (58) can collect 60 data points per second, compared to less than one data point per second typically collected from reaction time or choice tasks. One recent line of research has found that computational measures of dysfunctional real-time control processing can be estimated with very high split-half and test-retest reliability (57, 58) and are associated with both anxious arousal (57) and PTSD diagnosis (59) as well as activation of arousal-related brain regions during inhibitory processing (60). Given accumulating evidence that neural sensorimotor control represents a rich computational process influenced by neural arousal and reward mechanisms (56, 61, 62), a focus on sensorimotor tasks is a promising direction for computational psychiatry, especially as applied to elevated arousal. It will be important to develop markers which are both feasible and cost effective, especially for deployment in a variety of countries and healthcare systems.

Once reliable markers of a high arousal PTSD subgroup have been established, they can be incorporated into clinical trials of novel GABAergic agents for PTSD. The approach, in which objective markers are incorporated into clinical trials, is consistent with National Institute of Mental Health (NIMH) initiatives including experimental therapeutics (which examines the influence of interventions on mechanistic targets) (63) as well as the Research Domain Criteria (RDoC) (64), which seeks to move beyond symptom-based diagnostic categories. In addition to objective markers, clinical trials can incorporate self-reported symptoms into subgroup assignment.

In conclusion, recent research has emphasized the substantial individual heterogeneity within the diagnostic category of PTSD, suggesting that interventions targeting distinct mechanisms might be effective for specific subgroups of patients. One potential subgroup identified in studies based on self-reported symptoms as well as behavioral and biological markers is a group with a specific elevation in anxious arousal. In parallel, converging evidence from both causal and correlational studies suggests that novel GABAergic medications may be particularly effective in reducing anxious arousal. Taken together, these literatures suggest that novel GABAergic agents may be particularly effective in a high arousal PTSD subgroup. Future clinical trials of these agents should incorporate subjective and objective measures of anxious arousal to assess their effectiveness in specifically selected patients.
